# Impact of the type of anterior lamellar reconstruction on the success of modified Hughes procedure

**DOI:** 10.5935/0004-2749.20200011

**Published:** 2020

**Authors:** Meryem Altin Ekin, Seyda Karadeniz Ugurlu

**Affiliations:** 1 Department of Ophthalmology, Izmir Katip Celebi University Ataturk Training and Research Hospital, Izmir, Turkey

**Keywords:** Surgical flaps, Anterior lamella, Carcinoma, basal cell, Skin transplantation, Conjunctiva/transplantation, Retalhos cirúrgicos, Lamela anterior, Carcinoma basocelular, Transplante de pele, Conjuntiva/transplante

## Abstract

**Purpose:**

To determine the long-term functional and cosmetic outcomes in patients who underwent modified Hughes procedure with different types of anterior lamellar reconstruction for lower eyelid defects.

**Methods:**

This study included 58 patients who had undergone a modified Hughes flap for reconstruction of lower eyelids after tumor excision within a 10-year period. Data regarding patient demographics, size of eyelid defect, tumor pathology, surgical techniques, functional and cosmetic outcomes, and complications were recorded. Postoperative complications were evaluated according to the type of anterior lamella reconstruction (i.e., advancement flap or free skin graft). Multivariate logistic regression analysis was performed to identify risk factors affecting the success of the procedure.

**Results:**

The average size of the lower eyelid defect was 22 ± 6.3 mm (range: 11-30 mm). The anterior lamella was reconstructed with advancement flaps and full-thickness skin grafts in 36 (58.6%) and 24 (41.4%) patients, respectively. Mean follow-up time was 23.6 ± 11.9 months. Postoperative complications included trichiasis (three patients; 5.2%), ectropion (two patients; 3.0%), flap necrosis (one patient; 1.7%), flap dehiscence (one patient; 1.7%), infection (one patient; 1.7%), and eyelid margin erythema (one patient; 1.7%). The rates of complication and secondary surgery were similar among the different types of anterior lamellar reconstruction (p=768 and p=0.139, respectively). Success of the modified Hughes procedure was not significantly affected by any of the identified risk factors (p>0.05). Functional and cosmetic outcomes were 96.6% and 94.8%, respectively.

**Conclusion:**

Modified Hughes procedure is a safe and effective option for the reconstruction of small and large defects of the lower eyelid, regardless of the type of anterior lamella reconstruction (i.e., advancement flap or skin graft).

## Abstract (pt)

**Objetivo:**

Determinar os resultados funcionais e cosméticos a longo prazo de pacientes submetidos ao procedimento de Hughes modificado com diferentes tipos de reconstrução lamelar anterior para defeitos palpebrais inferiores.

**Métodos:**

Este estudo incluiu 58 pacientes que foram submetidos a um retalho de Hughes modificado para reconstrução das pálpebras inferiores após excisão do tumor durante um intervalo de 10 anos. Dados referentes à demografia dos pacientes, tamanho do defeito palpebral, patologia tumoral, técnicas cirúrgicas, resultados funcionais e cosméticos e complicações foram registrados. As complicações pós-operatórias foram avaliadas de acordo com o tipo de reconstrução da lamela anterior (ou seja, retalho de avanço ou enxerto de pele livre). A análise de regressão logística multivariada foi realizada para identificar os fatores de risco que afetam o sucesso do procedimento.

**Resultados:**

O tamanho médio do defeito da pálpebra inferior foi de 22 ± 6,3 mm (11-30 mm). A lamela anterior foi reconstruída com retalhos de avanço e enxertos de pele de espessura total em 36 (58,6%) e 24 (41,4%) pacientes, respectivamente. O tempo médio de acompanhamento foi de 23,6 ± 11,9 meses. Complicações pós-operatórias incluíram triquíase (três pacientes: 5,2%), ectrópio (dois pacientes: 3%), necrose de retalho (um paciente: 1,7%), deiscência de retalho (um paciente: 1,7%), infecção (um paciente: 1,7%) e eritema na margem palpebral (um paciente: 1,7%). As taxas de complicação e de cirurgia secundária foram semelhantes entre os diferentes tipos de reconstrução lamelar anterior (p=768 e p=0,139, respetivamente). O sucesso do procedimento de Hughes modificado não foi significativamente afetado por nenhum dos fatores de risco identificados (p>0,05). Resultados funcionais e cosméticos foram de 96,6% e 94,8%, respetivamente.

**Conclusão:**

O procedimento de Hughes modificado é uma opção segura e eficaz para a reconstrução de pequenos e grandes defeitos da pálpebra inferior, independentemente do tipo de reconstrução da lamela anterior (ou seja, retalho de avanço ou enxerto de pele).

## INTRODUCTION

The lower eyelid is characterized by its delicate and thin structure, and is highly susceptible to the development of skin cancer. Total excision of the lesion and reconstruction of the resultant defect is the recommended management for skin cancers involving the lower eyelid^(1)^.

The reconstruction approach depends on the location, size, and involvement of the anterior and/or posterior lamella in the defect. For full-thickness defects (i.e., <25% of the horizontal length of the eyelid), direct closure may be appropriate. For larger defects, repair of the lower eyelid may be more complex, including free grafts, rotational flaps, shared flaps, or a combination of these techniques^(2)^. Full-thickness de fects should be reconstructed in multiple layers to optimize cosmetic and functional outcomes. The eyelids may be divided into the following surgical units: anterior and posterior lamella. The anterior lamella consists of skin and the orbicularis muscle, while the posterior lamella consists of the conjunctiva and tarsal plate. Accordingly, both the anterior and posterior lamella should be reconstituted in full-thickness defects. Numerous procedures are available for the repair of large full-thickness lower eyelid defects, involving the use of the hard palate, nasal septal cartilage, or other free grafts to substitute the posterior lamella, combined with a transposition flap or cheek rotation flap for replacement of the anterior lamella^(3)^.

The Hughes tarsoconjunctival flap may be an effective alternative method for eyelid reconstruction^(4)^. Wendell Hughes originally described this procedure in 1937^(4)^. The incision of the classical Hughes procedure was initiated at the gray line of the lid margin, leaving the levator muscle aponeurosis and Müller’s muscle attached to the tarsal plate. A tarsoconjunctival flap from the upper eyelid was advanced to recreate the posterior lamella of the ipsilateral lower eyelid, and reconstruction of the anterior lamellar was performed using cheek skin. In the second stage (i.e., after 3-4 weeks), division of the pedicle was performed. In 1954, Macomber et al. used full-thickness skin graft harvested from either the postauricular, supraclavicular, or contralateral upper lid skin for the lower eyelid^(5)^. After 40 years of experience, Hughes developed a new technique by cutting obliquely through the tarsus, beginning at the conjunctival margin and extending to the anterior surface of the tarsus approximately 3 mm above the lid^(6)^. To prevent the occurrence of ipsilateral upper eyelid complications, further modifications were introduced. Cies and Bartlett suggested to leave the inferior portion of the upper eyelid tarsal plate *in situ* by performing the incision above the margin of the lid^(7)^. Another modification of the tarsoconjunctival flap procedure was the maximal Hughes procedure, combining oblique medial and lateral periosteal flaps with a Hughes flap for the repair of large defects of the lower lid^(8)^. The modified Hughes procedure described in the present study includes construction of the tarsoconjunctival flap 4 mm above the margin of the lid and transection of Müller’s muscle attachments at the superior edge of the tarsal plate. Notably, the dissection proceeds subadjacent to the conjunctiva.

The aim of this study was to determine the long-term functional and cosmetic outcomes in patients with lower eyelid defects who underwent modified Hughes procedure with different types of anterior lamellar reconstruction for lower eyelid defects.

## METHODS

This retrospective chart review study included patients who had undergone a modified Hughes flap for the reconstruction of the posterior lamella of lower eyelids after tumor excision within a 10-year period from 2008 to 2018 at Izmir Katip Celebi University Atatürk Training and Research Hospital, Izmir, Turkey. Patients with lid closure lasting several weeks, such as children at risk of occlusion amblyopia, patients with previous eyelid reconstruction, patients with a history of periocular radiotherapy, and those with monocular vision were excluded from this study. Medical records were reviewed to extract patient information, such as age, gender, indication for surgery, symptoms, methods of anterior lamella repair, horizontal width and surface area of the defect, histopathological diagnosis, follow-up interval, complications, and functional and cosmetic outcomes. The surface area of the defect was calculated by measuring the length, width, and height of the excised tissue using the following formula: SA = 2lw + 2lh + 2wh (l: length, w: width, h: height). All the patients with lower eyelid tumor were evaluated through a full ophthalmic examination involving visual acuity, ocular movements, anterior and posterior segment, and dry eye test. For histopathological diagnosis, patients underwent lesion biopsies. Following the detection of suspicious findings for invasion of surrounding structures, orbital magnetic resonance imaging was performed.

**Figure 1:**
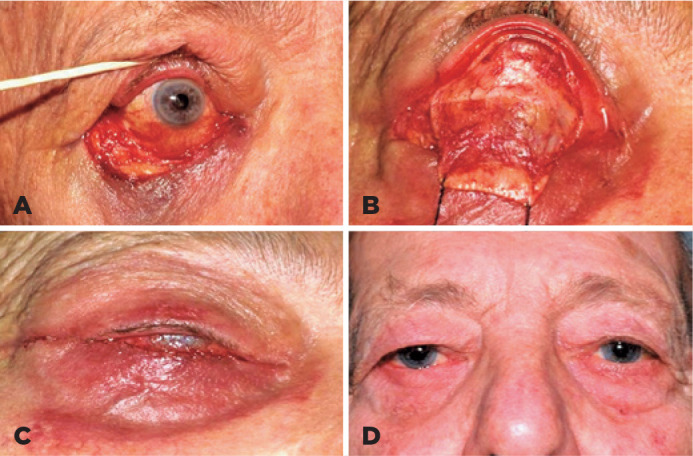
Lower eyelid defect following tumor excision (A). The defect was reconstructed using a modified Hughes tarsoconjunctival flap (B,C). Postoperative appearance of the same patient after division of the flap.

**Figure 2:**
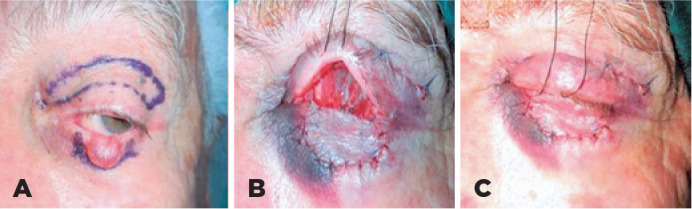
Skin marking of the full-thickness skin graft from the upper eyelid for the reconstruction of the ipsilateral lower eyelid after excision of basal cell carcinoma (A). Postoperative photograph of the same patient after undergoing a modified Hughes procedure with a free skin graft (B, C).

All the patients were informed regarding the reconstructive options and provided consent prior to the procedures. The modified Hughes procedure was performed by a single surgeon under general or local anesthesia depending on the preference of the patient. Following excision of the entire lesion in the lower eyelid, intraoperative histological evaluation of the tumor resection borders was repeatedly performed using frozen section, as required, until negative margins were achieved. The size of the defect was measured by pulling the medial and lateral boundaries of the eyelid wound toward each other using two pairs of forceps. In addition, the size of the flap to be constructed was ascertained. The upper eyelid was everted over a retractor. A horizontal incision was performed 4 mm above the margin of the upper eyelid, which corresponds to the width of the defect as measured before. The incision is deepened through the full thickness of the tarsus in an inverted U-shaped manner over the superior tarsal border by dissecting all Müller’s muscle attachments in the avascular pretarsal plane. The dissection continued in this plane between Müller’s muscle and the conjunctiva high into the fornix. This assisted in lowering only the conjunctiva with the tarsus, without significant tension to the posterior lamella of the upper lid. The tarsoconjunctival flap was sutured using a 6-0 absorbable polyglactin suture (Vicryl; Ethicon, Inc, Somerville, New Jersey, USA) to the medial and lateral cut tarsal edges, and to the lower eyelid conjunctiva inferiorly to complete the posterior lamellar repair (Figure 1). In patients with lateral canthal defects, a periosteal flap was raised from the lateral orbital rim to cover the lateral defect of the posterior lamella of the lower eyelid. The anterior lamella was created using an advancement flap or a free skin graft depending on the presence of adequate normal skin to drape over the tarsoconjunctival flap. When additional tissue was required, a semicircular rotational flap (Tenzel procedure) was used as described previously^(9)^. Free skin graft was harvested from the ipsilateral upper eyelid (Figure 2). The skin was sutured using 6-0 absorbable polyglactin sutures (Vicryl; Ethicon, Inc, Somerville, New Jersey, USA).

Prior to discharge from the hospital at the first postoperative day, wound healing was evaluated through inspection for signs of infection and suture integrity. The stitches were removed 1 week after surgery. The second stage was completed at 3-4 weeks postoperatively by slightly cutting the flap above the margin of the lower lid using scissors under local anesthesia. Postoperative follow-up was performed at 1 and 3 weeks, 1, 3, and 6 months, and every 6 months thereafter. During the follow-up, each case was examined for functional outcomes and postoperative complications included flap dehiscence, flap necrosis, flap pedicle rupture, hematoma, infection, symblepharon, entropion, lid retraction, ptosis, ectropion, lid margin hypertrophy, lid margin erythema, trichiasis, pyogenic granuloma, lagophthalmos, and tumor recurrence.

The cosmetic outcomes of the surgery were defined as satisfactory if the reconstructed lid did not exhibit lagophthalmos, contour irregularity, notching, unmatched color of the graft or flap, or noticeable scarring. Satisfactory cosmesis was judged based on patient satisfaction, as documented in the patient record at the last follow-up visit. Moreover, satisfactory cosmesis was also determined by the surgeon (S.K.U) using standardized follow-up photographs for each patient repeatedly captured 3 months after flap separation and thereafter. The functional outcomes were defined as normal by the surgeon (S.K.U) if the opening and closure function of the reconstructed lid was preserved, and lid malposition and lagophthalmos were not observed. Surgery was considered successful when a satisfactory cosmesis and a normal lid function were achieved without the requirement for additional surgical measures. Secondary operations for complications were also noted. Patients were grouped depending on the selected technique for reconstruction of the anterior lamella, and analyzed for the rate of surgical success and frequency of complications.

The SPSS version 20.0 software for Windows (IBM Corporation, Armonk, NY, USA) was used to perform statistical analysis. Data were expressed as the mean ± standard deviation for continuous variables, and number of cases and percentages for categorical variables. The Student independent *t* test was used to compare continuous variables. The chi-squared test was used to analyze categorical data. Multivariate logistic regression analysis was used to identify risk factors. A p<0.05 indicated statistical significance.

## RESULTS

Table 1 presents the demographic characteristics of the 58 patients included in this study. Of those, 35 (62.5%) and 14 (25%) patients were diagnosed with large (>50%) or complete eyelid defect, respectively. The anterior lamella was reconstructed using an advancement flap or a full-thickness skin graft in 36 (58.6%) and 24 (41.4%) patients, respectively. Additional procedures were utilized in addition to modified Hughes procedure when deemed necessary. The Tenzel procedure was used in 12 (20.7%) patients for reconstruction of the anterior lamellar. The periosteal flap was raised in 8 (14.3%) patients to provide fixation of the lateral canthal.

**Table 1:** Demographic characteristics of the study population

No. of patients	58
Age (years)	72 ± 11.4
Gender (M/F)	1.1
Horizontal width of the lesion (mm)	22 ± 6.3
Surface area of the lesion (mm^2^)	28 ± 10.4
Invasion to adjacent structures	2 (3.4)
Systemic metastasis	1 (1.7)
Histopathological diagnosis BCC see	51 (87.9) 7(12.1)
Canalicular involvement	6(10.3)
Lacrimal intubation	2 (3.4)
Method of anterior lamellar repair Advancement flap Free skin graft	34 (58.6) 24 (41.4)
Division of flap (days)	26.4 ± 10.1
Radiotherapy	2 (3.4)
Follow-up (months)	23.6 ±11.9
Cosmetic outcome Satisfactory Poor	55 (94.8) 3 (5.2)
Functional outcome Normal Limited Lost	56 (96.6) 2 (3.4) 0
Tumor recurrence	0
Complication	9(15.5)
Secondary surgery	4 (6.9)

Data are presented as the mean ± standard deviation or n (%). BCC= basal cell carcinoma; SCC= squamous cell carcinoma; M/F= male-to-female ratio.

The postoperative course was uncomplicated in 49 (84.5%) patients. In the early postoperative period, one (1.7%) patient presented with flap necrosis, while another patient (1.7%) presented with premature incomplete central dehiscence of the conjunctival pedicle. Wound infection was noted in one patient (1.7%) with poor personal hygiene at postoperative day 9. In the late postoperative period following flap division, two patients (3.4%) presented with lower eyelid ectropion, three patients (5.2%) with trichiasis, and one patient (1.7%) with erythema of the lower eyelid margin. The patient who developed the central dehiscence of the conjunctival pedicle recovered with good functional and cosmetic outcomes. Among the nine patients who developed a postoperative complication, four (44.4%) patients underwent secondary intervention for ectropion repair (two patients) and debridement (two patients). All the patients who underwent secondary repair exhibited satisfactory outcomes. Successful restoration of functional integrity was obtained in 56 (96.6%) patients, whereas it was limited in two (3.4%) patients. Cosmetic outcomes were satisfactory in 55 (94.8%) patients and poor in three (5.2%) patients.

Table 2 shows all postoperative complications related to the repair of the anterior lamella. Of note, there was no statistically significant difference regarding the rates of complications and secondary surgery between patients treated with an advancement flap or a free skin graft. A multivariate logistic regression analysis was conducted to determine the risk factors affecting the success of the procedure (Table 3). Age, gender, horizontal width and surface area of the defect, type of malignancy, type of anesthesia, type of anterior lamella reconstruction, time of flap division, and radiotherapy did not significantly affect the rate of surgical success (p>0.05).

**Table 2:** Postoperative rates of complications according to the type of anterior lamellar reconstruction

Type of complications	Advancement flap (n=36)	Free skin graft (n=24)	Total (n = 58)	pvalue
Early (prior to flap division)				
Flap dehiscence	-	1(4.2%)	1 (1.7%)	0.217
Flap necrosis	1 (2.8%)		1 (1.7%)	0.410
Infection	-	1 (4.2%)	1 (1.7%)	0.217
Late (after flap division)				
Lower eyelid				
Ectropion	-	2 (8.3%)	2 (3.4%)	0.078
Lid margin erythema	1 (2.8%)		1 (1.7%)	0.410
Trichiasis	3 (8.3%)		3 (5.2%)	0.146
Total complications	5 (13.9%)	4(16.7%)	9(15.5%)	0.768
Secondary surgery	1 (2.8%)	3 (12.5%)	4 (6.9%)	0.139

**Table 3:** Multivariate logistic regression analysis of the risk factors affecting success of the modified Hughes procedure

Variables	Odds ratio	95 % Confidence interval	p-value
Age >70 years	0.69	0.57-1.30	0.273
Female gender	1.22	0.92-1.67	0.311
Horizontal width of the lesion ≥15 mm	0.83	0.79-0.87	0.096
Surface area ≥15 mm^2^	0.60	0.49-1.61	0.103
Pathology of the lesion			
BCC	2.31	0.86-5.84	0.325
SCC	1.65	0.85-3.66	0.216
General anesthesia	1.20	0.94-1.58	0.244
Additional procedures			
Canalicular involvement	1.58	0.92-3.24	0.235
Lacrimal intubation	1.30	0.75-2.18	0.369
Tenzel flap	1.12	0.89-1.44	0.214
Periosteal flap	1.25	0.71-2.09	0.176
Type of anterior lamellar reconstruction			
Advancement flap	2.55	0.84-5.37	0.139
Free skin graft	1.39	0.74-4.23	0.168
Division of flap >21 days	2.59	0.88-4.68	0.362
Radiotherapy	0.91	0.65-2.14	0.455

BCC= basal cell carcinoma; SCC= squamous cell carcinoma

## DISCUSSION

This study included patients who suffered a lower eyelid malignancy and underwent surgery for reconstruction of the lower eyelid using the modified Hughes tarsoconjunctival flap. It was shown that the modified Hughes procedure is a suitable and valuable method for the reconstruction of major lower eyelid defects.

Numerous surgical approaches for the reconstruction of posterior lamella are currently available, such as free tarsal graft, hard palate graft, nasal septal cartilage, donor sclera, and periosteal graft^(3)^. Hughes tarsoconjunctival flap for reconstruction of the posterior lamella is more simplified and provides an improved blood supply compared with these methods^(8)^. However, recently, there has been some controversy regarding the blood supply of the Hughes flap. A study performed by Memarzadeh et al. in pigs showed that blood flow and tissue oxygenation were gradually decreased during dissection and advancement of the tarsoconjunctival flap^(10)^. Tenland et al. monitored perfusion in tarsocon-junctival flaps in patients with large lower eyelid defects resulting from tumor surgery^(9)^. They found that the blood flow was gradually decreased from the pedicle base to the end of the Hughes tarsoconjunctival flap^(11)^. Interestingly, flap survival was not compromised in neither of the studies^(10^,^11)^. Furthermore, it was possible to avoid extensive surgical procedures (e.g., nasolabial flap, midforehead flap, or cheek rotation flap) for the reconstruction of the anterior lamella. In this study, the anterior lamella was reconstructed using a skin advancement flap and free skin graft in 58.6% and 41.4% of patients, respectively. We did not observe statistically significant differences in the rates of surgical success and complications between the types of anterior lamella reconstruction. To the best of our knowledge, this is the first study performing a statistical comparison of these reconstructive techniques in the context of Hughes procedures in consecutive patients.

Due to cicatricial and mechanical causative factors, most lower eyelid reconstructive procedures are associated with a high incidence of ectropion^(2^,^3)^. In the present study, two patients had ectropion at 25 and 31 days postoperatively. The rate of ectropion (3.4%) observed in patients who underwent the modified Hughes procedure was lower than those reported for other reconstructive surgeries of the lower eyelid. In the study performed by Hawes et al., 15% of patients who received a free tarsoconjunctival flap for the repair an anterior lamellar defect developed lower eyelid ectropion^(12)^. Perry et al. noted that ectropion of the lower eyelid occurred in 5.2% of cases with lateral stabilization using a periosteal strip and myocutaneous advancement flap^(13)^. Similarly, we used this technique in 8 (14.3%) of the patients in this study^(13)^. However, none of our patients with periosteal flap developed ectropion during the postoperative period. It is proposed that upward vertical traction on the lower eyelid counteracts postoperative inferior vertical contraction. Ectropion occurred in patients who underwent modified Hughes procedures, in which free skin grafts were used for reconstruction of the anterior lamella. Those patients exhibited a horizontally oversized flap. Notably, it is more likely that eyelid retraction may occur due to horizontal lower lid laxity rather than gravitation pull of the anterior lamella.

Mustarde reported that loss or contraction of even a small part of the upper eyelid may result in exposure of the cornea and subsequently corneal ulceration^(14)^. He stated that there is no justification for compromising the structural integrity of the upper eyelid in an attempt to reconstruct the lower eyelid. For this reason, he suggested using other reconstruction methods of the lower lid instead of the Hughes flap^(14)^. However, thus far, we have not encountered the any upper eyelid complication including ptosis, entropion or retraction during the long-term follow-up period. Upper eyelid complications are more commonly observed in patients undergoing the classical Hughes procedure, in which the incision is initiated at the lid margin eventually splitting the upper eyelid in a posterior and anterior lamella. This results in the attachment of the levator and Müller’s muscle complex to the tarsus. The low incidence of upper eyelid complications reported in this study may be attributed to the modification of the Hughes procedure. In this modification, the inferior edge of the flap was designed at 4 mm from the margin of the lid and the attachments of Müller’s muscle were transected at the superior edge of the tarsal plate. Subsequently, the dissection was performed upward along a plane adjacent to the conjunctiva. Consequently, a tarsoconjunctival flap was developed by dissecting the tarsus and conjunctiva away from the levator aponeurosis and Müller’s muscle. Thus, sparing of the marginal upper lid tarsus and the removal of the Müller’s and levator muscle complex from the tarsoconjunctival flap may reduce the occurrence of upper eyelid complications (e.g., ptosis, entropion, or necrosis of the eyelid margin).

Other adverse outcomes noted in the present study are trichiasis (n=3), flap dehiscence (n=1), flap necrosis (n=1), graft infection (n=1) and erythema of the eyelid margin (n=1). Postoperative flap necrosis and graft infection in the anterior lamella, requiring surgical debridement and antibiotic treatment, developed at 7 and 9 days, respectively. Further potential complications included pyogenic granuloma and the development of an eyelid margin cyst, which did not occur in our study population. Bartley and Messenger reported a 12.5% incidence of premature flap dehiscence within 11 days after undergoing a modified Hughes procedure^(15)^. However, flap dehiscence - which occurred in seven of eight patients - was caused by accidental trauma. In spite of this, the investigators suggested that a dehiscent Hughes flap does not necessarily require repair due to favorable outcomes^(15)^.

The different types of grafts or flaps used for the repair of lower eyelid defects have demonstrated an average complication rate of 38.6%^(11)^. In the present study, 15.5% of patients developed a postoperative complication. In 6.9% of patients, these complications were judged by the patient or physician to require further intervention. The success of the modified Hughes flap is comparable to that reported for other techniques used for the reconstruction of lower lid defects, while preventing the occurrence of numerous complications observed with these techniques. Moreover, the rate of patient satisfaction reported in this study was comparable to that shown in other studies using the Hughes flap. Engelmann et al. reported that 92.3% of the patients undergoing a Hughes flap were subjectively satisfied or even very satisfied with the esthetic outcome^(16)^. The present patients demonstrated excellent esthetic and functional outcomes in the long-term follow-up. This approach results in satisfactory cosmesis and normal lid function without the requirement for further surgery in >90% of patients.

Another factor that may affect the success of the modified Hughes procedure was the horizontal width of the lesion. Although not reached, there was a tendency toward statistical significance (OR, 0.83; 95% CI, 0.79-0.87; p=0.096). In addition, previous studies have revealed a significant relationship between the horizontal width of the lesion and success of the modified Hughes procedure^(12^,^17)^. However, our study differs from previous investigations in that it explored favorable outcomes in the reconstruction of large lower eyelid defects using the modified Hughes procedure. Notably, the mean horizontal width diameter observed in the present study was longer than those reported in most of the previous studies (i.e., average diameter <20 mm)^(18^-^20)^. This finding demonstrated that the size of the reconstructed defects did not affect the cosmetic and functional outcomes. For the treatment of large defects involving the lower eyelid, the modified Hughes technique may be the procedure of choice.

A disadvantage associated with the Hughes procedure is it involves two stages, rendering the patient monocular for a period of time due to eyelid closure on the affected side. Monocular vision may be a problem for children at risk of developing amblyopia and those with monocular vision prior to intervention. In our study, the average timing of pedicle division was 26.4 days after primary operation. However, in patients in whom monocular vision is not desired, the tarsoconjunctival pedicle may be safely divided earlier without an increase in the risk of eyelid malposition or complications. More recent studies have demonstrated good functional and cosmetic outcomes with early division of the tarsoconjunctival pedicle of a modified Hughes flap at 1 week^(19)^. Additionally, one-stage techniques that do not render the patients temporarily monocular have been proposed as alternatives to the Hughes procedure. Skippen et al. employed three different techniques and reached a 94% patient satisfaction rate without occurrence of ectropion, hyperemia of the eyelid margin, flap ischemia, necrosis, or failure^(17)^. However, 33% of patients (12/36) developed other complications, such as lanugo hair distichiasis (19%), eyelid margin skin cyst (6%), pyogenic granuloma (3%), entropion (3%), and retraction of the lower eyelid (3%)^(17)^.

A limitation of this study is its retrospective nature. Moreover, a control group to compare the alternative techniques to Hughes procedure was not included in this study. However, the size of the study population and the long follow-up period versus those previously reported in the literature are the strengths of the present investigation.

In conclusion, a modified Hughes technique was successfully utilized for the reconstruction of both small and very large defects involving the lower eyelid. This is a safe and simple method, providing a lid of acceptable function and appearance. It was also demonstrated that use of the advancement flap or free skin graft in the modified Hughes procedure offers similar results in the reconstruction of the anterior lamella of the lower eyelid.
